# Detrusor underactivity: To tone or not to tone the bladder?

**DOI:** 10.4103/0970-1591.56186

**Published:** 2009

**Authors:** Sriram Krishnamoorthy, Nitin S. Kekre

**Affiliations:** Department of Urology, Christian Medical College, Vellore, Tamil Nadu, India

**Keywords:** Detrusor underactivity, Bethanechol chloride, parasympathomimetics

## Abstract

**Objectives:**

The aim of this review was to review the available evidence in literature for the clinical effectiveness of Bethanechol Chloride in patients with detrusor underactivity.

**Materials & Methods:**

We searched all relevant data from Medline and peer-reviewed journals available online on the use of bethanechol in patients with detrusor underactivity.

**Results:**

Most reports that suggest a therapeutic clinical benefit with use of bethanechol have all been anecdotal reports and there is no definite clinical evidence available till date to support its clinical usefulness.

**Conclusion:**

The current available data have shown to offer no definite benefit with the use of parasympathomimetic agents in patients with DU. One of the meta-analysis has shown bethanechol to be ineffective in promoting bladder emptying.

## BACKGROUND

Detrusor underactivity (DU) is a common but highly under diagnosed geriatric condition. The clinical diagnosis is often difficult as the symptoms are non specific and can be diagnosed only by urodynamics.[1] The clinical settings where one encounters the problem of DU are: (i) diabetic bladder, (ii) chronic retention of urine, and (iii) neuro-vesical dysfunction. Some have advocated the use of parasympathomimetic drugs like bethanechol chloride (BC) for the treatment of underactive detrusor with a view to improve the bladder contraction. Are these drugs really beneficial? Is there scientific evidence available to support the efficacy and safety of this drug in the treatment of DU? A literature search has been performed by Pubmed using the key words detrusor underactivity, hypo-contractile detrusor, chronic retention of urine, and bethanechol chloride. This evidence-based review is undertaken to answer the above questions and to assess the quality of evidence to support the use of this drug.

## DETRUSOR UNDERACTIVITY

DU is defined as reduced strength and/or duration resulting in prolonged bladder emptying and/or a failure to achieve complete bladder emptying in a normal time span.[1] It can occur as a result of either a primary lack of stimulus for detrusor contraction or secondary to defective tissue responsiveness. The primary stimulus for detrusor contraction is acetyl choline, which acts on muscarinic (M3) receptors of the bladder musculature. Lack of acetyl choline can lead to a defective contraction of the bladder musculature, resulting in detrusor underactivity.[2]

With ageing, the cholinergic mechanism becomes defective while the neural pathway and the bladder musculature still remain normal. There is evidence to suggest that such patients might derive some benefit from BC. In cases of muscle loss or axonal degeneration, these drugs will not be effective.[1]

## EVIDENCE-BASED ANALYSIS

No randomized trials have been conducted to support the use of parasympathomimetics in DU. Barrett, in a double-blind, placebo-controlled, randomized trial studied the effects of oral BC on voiding in patients with chronic retention of urine. No differences in voided volumes, residual volumes, or mean flow rates were observed between the treated and control groups.[3] Of the 13 patients who had complaints of headache, abdominal cramps, and flushing, 9 patients had taken an oral dose of 50 mg of BC or more. They postulated that the side effects might be due to a higher dosage of the drug and that the effects of BC on trigone and bladder neck might also result in an increased frequency of micturition and an increase in the outflow resistance in these patients. Hindley, *et al*. used oral BC with intra-vesical prostaglandin (PGE2). In their prospective double-blind randomized trial of 19 patients with DU, only 4 out of 9 patients receiving active drug were shown to have symptomatic improvement and were able to reduce the frequency of clean intermittent self catheterization.[4] There were no serious adverse events and all 19 patients could complete the course of treatment. However, the therapeutic effect of this combination had been found to be of limited benefit when compared with the placebo combination.

Diabetes mellitus is the most common cause of sensory dysfunction of the urinary bladder, resulting in sensory and autonomic poly-neuropathy. This results in an impaired sensation of bladder fullness, increased bladder capacity, reduced detrusor contractility, and an increase in the residual volume.[5] BC in such conditions cannot be expected to show any benefit as the primary problem is not a lack of parasympathomimetic agent, but an inherent sensory and a myogenic failure.

Barendrecht, *et al*. performed a meta-analysis on the efficacy of parasympathomimetics for underactive detrusor. They analyzed ten published studies that used BC, Carbachol, or Distygmine.[6] They concluded that there is little evidence to support the use of parasympathomimetics or acetyl cholinesterase inhibitors in the treatment of DU. The reason for a poor therapeutic response can either be because of the unresponsiveness of the detrusor towards a contractile stimulus as in cases of post pelvic surgeries or because the dosage that is given is too low to induce a therapeutic response. The higher doses of BC produce a slightly higher benefit, but at the cost of enhanced side effects, preventing their further use.

Kemp, *et al*. supported the claim that BC is beneficial for patients who had undergone Wertheim's hysterectomy, but the number of patients included in this study were too small to derive any valid conclusion.[7] Riedl and his colleagues administered BC in the form of electromotive intra-vesical instillation.[8] In their study of 45 patients with DU, they observed that in patients who had an electromotive instillation, the penetration through urothelium onto the bladder wall was enhanced and elicited a better detrusor contraction. Such a benefit was observed in only a select group of patients.

## CONCLUSIONS

The current available data have shown no definite benefit with use of parasympathomimetic agents in patients with DU. Moreover, the presence of a pharmacological activity alone should not be considered tantamount to clinical or therapeutic efficacy. Most of the reports that suggest a clinical usefulness of BC have all been anecdotal reports, with no definite clinical evidence to suggest its therapeutic efficacy. The meta-analysis by Barendrecht and his colleagues has clearly shown BC to be ineffective in promoting bladder emptying, regardless of the dosage and route of administration.
